# Radiologic Evaluation of Chronic Vertebral Compression Fractures and Role of Vertebral Augmentation

**DOI:** 10.7759/cureus.3208

**Published:** 2018-08-27

**Authors:** Jesse Hatgis, Ovidiu Palea, Yashar Ghomri, Michelle Granville, Aldo Berti, Robert E Jacobson

**Affiliations:** 1 Pain Management, Phoenix Neurological and Pain Institute, Chandler, USA; 2 Anesthesiology and Pain Management, Centrul De Diagnostic Si Tratament Provita, Bucharest, ROU; 3 Pain Managment, Nova Southeastern University, Los Angeles, USA; 4 Miami Neurosurgical Center, University of Miami Hospital, Miami, USA; 5 Neurosurgery, University of Miami Hospital, Miami, USA

**Keywords:** vcf, chronic vertebral compression fractures

## Abstract

The literature has classified chronic vertebral compression fractures (VCF) as those still "symptomatic" four or more months after onset. Pain is regarded as the predominant chronic symptom; however, radiologic changes are important in evaluating fracture progression. This review examines a series of patients with chronic fractures and both persistence of spinal pain combined with radiologic changes, such as worsening collapse, spinal angulation, the development of vertebral edema and clefts, as well as the development of new fractures at adjacent spinal levels. In patients with clear progressive radiologic changes in addition to pain, vertebral augmentation on an average of 9.3 months after injury was effective in reducing the pain and stabilizing these more chronic osteoporotic fractures. A comparison of the pre- and post-procedure visual analog scale score (VAS) indicated an average of 66% reduction in pain. There are several reasons for the development of chronic symptomatic fractures. Most commonly, interventional treatment is delayed in a patient already diagnosed with VCF after a long period of conservative treatment, yet pain persists, or the initial clinical and radiologic evaluation misses the fracture, leading to a delay in diagnosis and treatment. In this report, management in these patients and the role of late vertebral augmentation for chronic symptomatic fractures is clarified based on the findings of various radiologic changes seen on both initial and follow-up radiologic studies.

## Introduction

The use of vertebroplasty (VP), vertebral augmentation (VA), or kyphoplasty (KP) to treat osteoporotic vertebral compression fractures (VCF) and the timing of these procedures is debated in the literature since the majority of patients resolve their initial pain and often go on to fracture healing with conservative treatment, including initial bracing and later spinal extension exercises and the medical management of the underlying osteopenia and osteoporosis [1]. The timing of an interventional procedure, such as VA or KP, has been studied, grouping acute and subacute fractures as those treated within the first three months after the initial injury, compared to more chronic fractures, which are defined as being seen or treated more than four months, or 16 weeks, since the onset of symptoms [2-3]. Interventional treatment has been reported to be effective as long as 24 months after onset for chronic pain secondary to VCF [4]. However, reports on the treatment of more chronic fractures have not specifically addressed the radiologic findings that may indicate in which cases late VA or KP may be effective although it is clear that patients can get significant pain relief [4-6]. Once clinical and radiologic findings confirming the presence of a chronic VCF are made, there is controversy about how long after the injury that interventional treatments, such as VA and KP, can still be effective [1-3]. If there is a significant delay in diagnosis or a missed diagnosis of persistent pain after a long period of conservative treatment, the question arises if treating the fracture with VP or KP is still effective in relieving chronic pain in selected patients. This review will examine a group of 31 patients from three different centers with chronic fractures that were treated with late vertebroplasty or vertebral augmentation at least four months after the original onset of the fracture. The study will also examine the distribution of these fractures and radiologic characteristics on magnetic resonance imaging (MRI) and computed tomography (CT) scan as well as associated kyphosis and scoliosis.

## Materials and methods

This multicenter review covered three centers over an 18-month period. Charts were reviewed identifying patients treated with vertebral augmentation or kyphoplasty four or more months after the onset of symptoms. The review of the patients included age, sex, previous history of osteoporosis and fractures, levels, symptoms, pre-procedure visual analog scale (VAS) score and post-procedure VAS score. Radiologic findings were reviewed on plain X-rays, bone scans, computerized tomography (CT) and magnetic resonance imaging (MRI). The level of fracture(s), the percentage of vertebral body collapse on CT or MRI scan, the degree of kyphosis or scoliosis, if present, the presence of edema or vacuum changes at the vertebral endplate or within the disc space, and the existence of vertebral clefts on MRI scan was noted. The choice of procedure was made by the individual surgeon. Radiologic findings, such as lumbar or thoracic scoliosis, spondylosis, and, specifically, the presence of lumbar spinal stenosis or spondylolisthesis were noted.

The surgical technique, performing vertebral augmentation versus kyphoplasty, and if the procedure was unilateral or bilateral was decided by the individual surgeon. All procedures were performed as outpatient procedures under local anesthesia with minimal sedation, as necessary. Different systems and types of bone cement were used, including Stryker (Malvern, PA, US), Medtronic (Minneapolis, Minnesota, US), and Renovaspine-Biopsybell (Mirandola, Italy). The cement used was either polymethylmethacrylate (PMMA) or bioactive calcium phosphate micro-glass cement (Cortoss, Stryker, Malvern, PA, US).

## Results

Combining retrospective chart reviews from the three centers, 31 cases were identified that were treated with vertebral augmentation for painful chronic fractures. The patients were treated from four to more than 36 months after injury, averaging 9.3 months. There were two cases treated four and five years after onset. The average age was 76, ranging from 56 to 90 years of age, and females made up 83% of the patients. The radiologic diagnosis was made by plain radiographs, bone scans, and CT and MRI scans. Since this was a retrospective study, there was an inconsistency on what radiologic study was used to compare the initial fracture with later studies before treatment.

The distribution of the locations of the fractures was similar in all three centers. A tabulation of the distribution found that lumbar fractures accounted for 60% of the fractures while thoracic fractures made up only 22%. This is actually the reverse of the normal distribution of VCF, where thoracic and thoracic-lumbar fractures make up between 40%-55% of all fractures and lumbar fractures only make up 20%-30% of the total. Since the average age of the patients was 76, not unexpectedly, MRI and CT scans revealed that over 50% of patients also had radiologic findings of degenerative lumbar spondylosis as well as lumbar scoliosis, stenosis, or spondylolisthesis. The fact that lumbar fractures were found in a higher incidence than the normal distribution of osteoporotic fractures may indicate that the original complaint of pain, especially if in the lumbar area in elderly patients, can be confused or masked by the underlying lumbar degenerative disease [7-9] (Table 1).

**Table 1:** Tabulation of fracture levels

LEVEL	ALL FRACTURES
T7	2
T8	2
T11	1
T12	4
L1	4
L2	2
L3	4
L4	6
L5	5
SACRUM	4

In attempting to determine the most informative study, we looked at the use of plain radiographs, CT, MRI, and bone scans. Plain radiographs and CT scans showed an average pre-procedure decrease in sagittal vertebral height of 60% at the time of vertebral augmentation compared to 20%-40% at the time of initial diagnosis. Progressive kyphosis of at least 10 degrees was found in 35%. Interestingly, the majority of the kyphotic deformities were found at the thoracic-lumbar junction. MRI scans demonstrated vertebral edema in 87% and vertebral clefts and vacuum endplate changes on CT and MRI scans were found in 23%, primarily located in the thoracic-lumbar junction. Bone scans were performed when there was a question of delineating possible "age," especially when there were multiple fractures or existing imaging studies demonstrated a fracture but without MRI edema or a vertebral cleft. Patients often had multiple different but confirmatory radiologic findings, such as vertebral edema and a vertebral cleft on MRI or the progressive collapse of vertebral height on follow-up films (Table 2). Bone scans were performed in 17 patients and were positive in 14, or 82%; however, one center did not do bone scans. The pre-procedure VAS score was 8.5 and the three-month, follow-up post-procedure dropped to 2.6. This is consistent with other reports of treatment of chronic VCF that looked at both immediate and long-term follow-ups and found a similar reduction in VAS scores [4-6]. This reduction in VAS score by 66% at three months included five patients whose follow-up VAS was 0, indicating that in the properly selected chronic VCF patient, vertebral augmentation is very effective.

**Table 2:** Breakdown of radiologic changes MRI: magnetic resonance imaging

LEVEL OF FRACTURES	ALL FRACTURES	SINGLE
% Thoracic fractures (#6)	33%	38%
% Thoraco-lumbar fractures (T12-L1) (#9)	21%	31%
% Lumbar fractures (#21)	60%	54%
% Sacral fractures (#4)	7.3%	7.7%
Average % vertebral collapse	62%	44%
% patients with kyphosis >10^o^	29%	23%
% patients with MRI edema at least at one fracture level	57%	77%
% patients with vertebral cleft on MRI	24%	38%
% patients with lumbar scoliosis	43%	20%
% patients with radiologic evidence of lumbar stenosis or degenerative spondylolisthesis on MRI	57%	51%

## Discussion

Clinical symptoms and radiologic evaluation

The decision for choosing different treatment options for VCF is based on both clinical symptoms, which is usually localized spinal pain after a fall or minor injury to the back, matched with concurrent radiologic findings in osteoporotic patients [7-8]. Although localized pain is the predominant initial clinical complaint of thoracic and lumbar VCF, the patient's pain can change and become more generalized over months. Thoracic fractures are most frequent but complaints of pain noted in the upper lumbar and lower thoracic spine may indicate a thoracic or thoracic-lumbar VCF, and fractures in this area can lead to a more flexed position (kyphosis) on examination [8-9]. Sacral fractures are frequently missed because of the vagueness of the pain, and they can also present with indirect symptoms, such as pain in the hip and groin, rather than in the low lumbar spine and sacrum [10-11]. Since VCFs occur in an older population, these same patients often have concurrent lumbar degenerative osteoarthritis, spondylolisthesis, and stenosis, so care needs to be taken in reviewing symptoms as well as radiologic studies for lumbar pain after a minor fall or accident [12]. Patients with previous lumbar surgery and fusions, especially with spinal instrumentation, are more prone to developing a VCF above the fusion, which may not be initially recognized, as attention is focused on the previous lumbar surgery [13]. In these cases, it will be the persistence of pain despite physical therapy or a recognition that the pain is in a different location than the lower lumbar spine in combination with new radiologic tests demonstrating a VCF that leads to the fracture being recognized as a cause of the pain.

Radiologic studies of VCF

Initial imaging studies, as well as follow-up studies combined with localized pain, are key in evaluating a patient with a possible symptomatic chronic VCF. There is a 20%-30% incidence of multiple fractures, often in other regions of the spine, that may have occurred previous to the current injury or at the same time, so initial films should always include both the thoracic, thoracic-lumbar junction, and lumbar spine even when the patient presents for the first time with pain localized only in one region [8-9]. The presence of previous or multiple sequential fractures is significant and changes both the immediate treatment, as well as the long-term treatment and general prognosis, including life expectancy [1,4]. It is important to make sure that the entire symptomatic area is included in the radiologic field and adjacent areas, such as the thoracic-lumbar junction, are adequately visualized to avoid missing junctional fractures [14-15]. Poor-quality plain X-ray films can occur because of technician error, patient obesity, and especially because of the poor bone detail frequently seen in osteoporotic vertebrae. Osteoporosis combined with underlying degenerative spinal scoliosis may distort the visualization of the spine and "hide" a fracture. In cases with inadequate preliminary imaging due to obesity or poor bone calcium with severe osteoporosis, CT or MRI scanning may be necessary to make a definitive fracture diagnosis. Fractures still can be missed at the edge of even good-quality films, whether examining plain X-rays, computerized tomography (CT), or magnetic resonance imaging (MRI).

**Figure 1:**
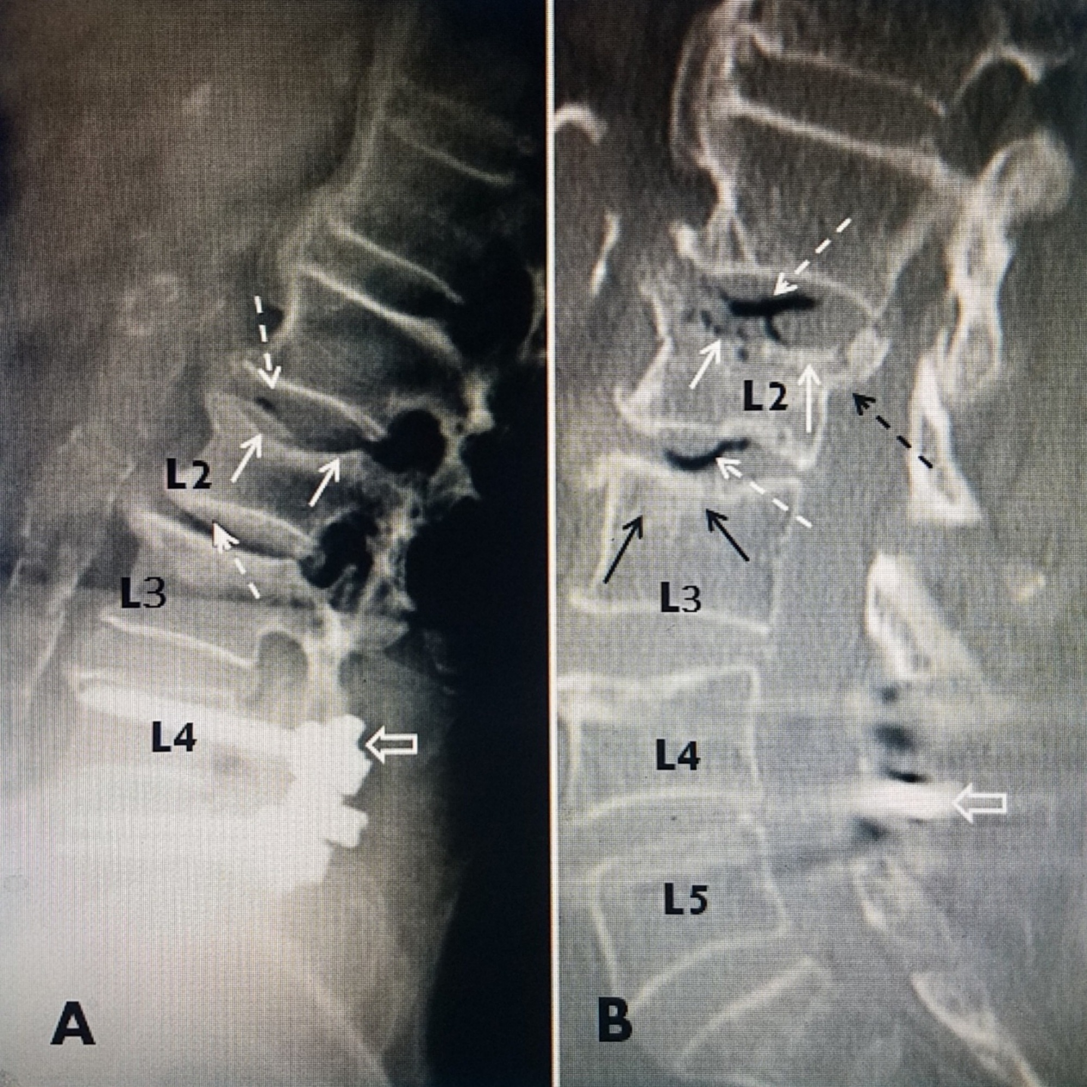
Vacuum change in the disc space surrounding an unstable fracture A: A 68-year-old female with a plain X-ray four weeks after a fall; plain radiograph showing a superior endplate fracture at L2 (solid white arrow) and previous pedicle screw fixation L4-5 (open white arrow). There is a subtle vacuum change at the L1-2 and L2-3 disc spaces (dashed white arrow). B: Because of persistent upper lumbar pain after six months conservative treatment, sagittal reconstruction of computerized tomography (CT) was performed and now clearly shows extensive vacuum changes at the L2-3 and L1-2 disc spaces (dashed white arrows). There is now a broader L2 superior endplate collapse with irregular interruptions in the continuity of the superior endplate (solid white arrows). On the CT, there is clearly the visualization of a more extensive posterior displacement of the superior endplate of L2 (dashed black arrow) into the ventral spinal canal. Part of the L4 screw is indicated by the open white arrow.

Initial films are assessed for level of fracture, percentage of collapse, and any degree of angulation or kyphosis. Scoliosis is not commonly caused by VCF but may be present secondary to underlying spinal degeneration and may complicate film interpretation and, later, any interventional procedure leading to poor pain relief or failure of vertebral augmentation [15]. A radionucleotide bone scan, showing one or more areas of positive vertebral uptake, is an important sign of fracture activity. However, bone scans are reported to have some degree of uptake as long as 24 months after a fracture without symptoms. Bone scans are able to show a fracture but not necessarily if it is related to the patient's pain. Bone scans can also detect other unsuspected vertebral fractures in an area with a documented vertebral fracture. Bone scans often detect fractures in ribs, the clavicle, or the long bone areas associated with a fall, even though the primary reason for the scan was localized spinal pain [16]. In patients with single-level fractures, there is a high correlation between the location of pain and uptake on the bone scan, but in patients with multiple fractures, the correlation is weaker so the disparity between the clinical area of pain and uptake in the bone scans becomes problematic with multiple fractures so correlation between symptoms, plain X-rays, bone scan, CT, and MRI may be needed [17]. Nevertheless, a negative bone scan, especially with multiple fractures, does not exclude seeing edema on MRI, indicating a possible non-healed fracture [17]. When there is a time interval extending weeks or months after the initial acute injury, it is difficult to determine if the fracture seen on radiologic studies is healing or still "active" and actually the source of pain. It is also possible to have MRI evidence of vertebral edema and bone scan uptake, but without a clear vertebral collapse in a patient with chronic pain so it is the constellation of findings related to clinical pain that may be important in evaluating more chronic vertebral compression fractures [18]. As a longer time interval passes from the initial injury with an untreated fracture, the patients are also at risk to develop other fractures secondary to the underlying osteoporosis. This is especially common with thoracic and thoracic-lumbar fractures that have a kyphotic deformity with a shift of the center of gravity more anteriorly [18]. This, in turn, leads to further load-bearing on already weakened, fractured vertebra, leading to progressive fracture and collapse, such as vertebrae plana as well as the development of fractures in adjacent vertebrae, so interventional treatment without updated imaging may lead to missing evolving fractures [14]. In the case of a chronic lumbar VCF, there is often deformity from degenerative scoliosis and stenosis, which can complicate identification as well as treatment of the fractures. Vacuum changes or phenomena in the disc space can be seen both with degenerative disc disease and VCF but acute worsening is more typical with chronic fractures [19]. In all of these cases, selected repeat radiology studies, usually CT or MRI scans, before any decision on treatment is important to avoid missing the progression or development of other fractures that could have evolved from the injury. Planned interventional treatment CT and MRI scans are more accurate for measuring the degree of collapse, the presence of a "vacuum" change near the endplate or in the intervertebral disc space (Figure 1).

**Figure 2:**
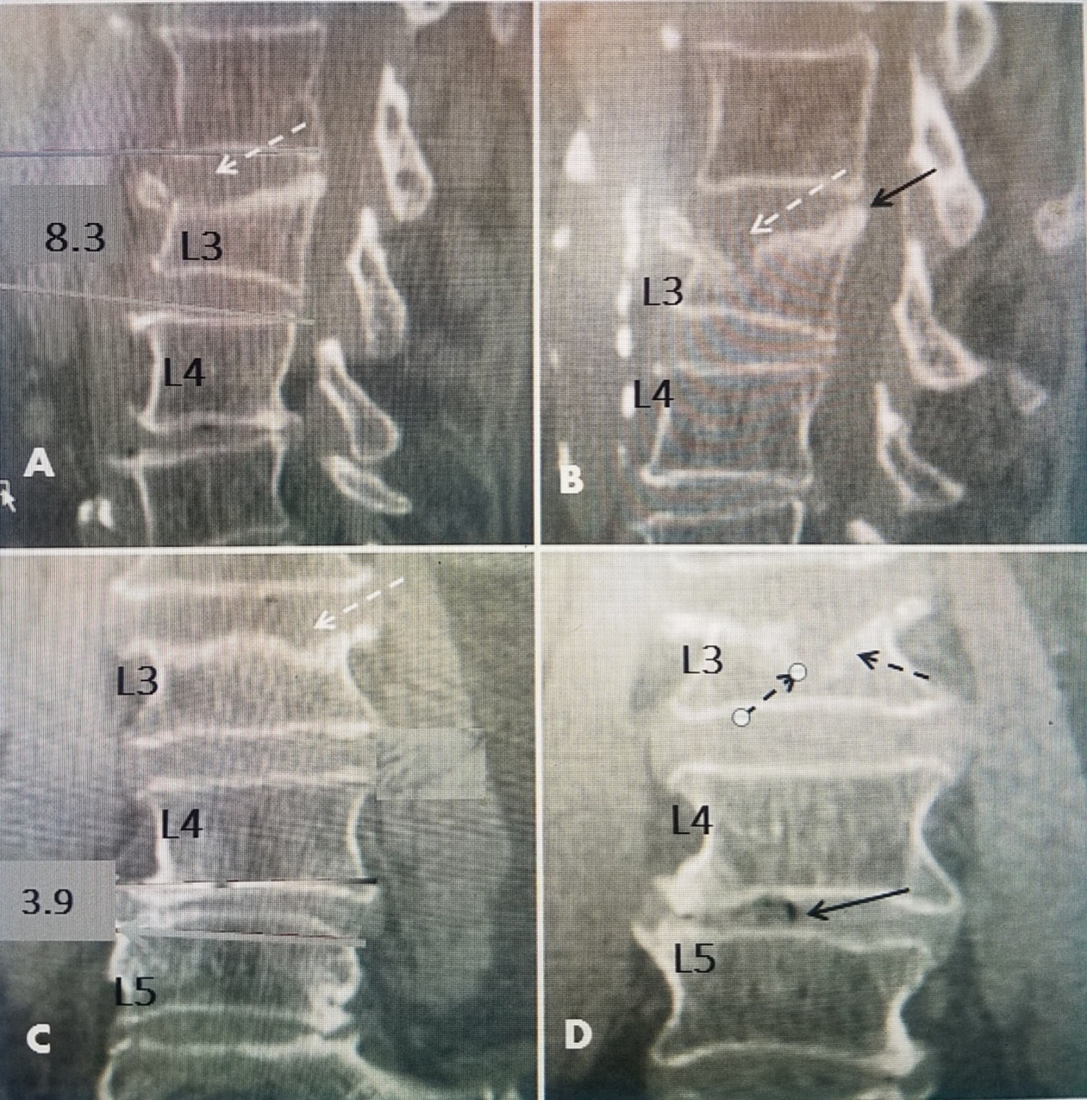
Computerized tomography (CT) scan of a chronic fracture nine months after injury A 76-year-old female with known osteoporosis and bone mineral density (BMD) of -2.3 in the lumbar spine. Initial and follow-up CT scans made nine months after the fall. The patient persisted with chronic mid-lumbar pain despite six months of lumbar bracing. Visual analog score (VAS) of 9. A: Initial CT scan 10 days after fall, showing a 20% anterior superior endplate collapse of L3 (dashed white arrow) with 8.3^0^ of kyphotic angulation and no displacement of the endplate of L3 into the spinal canal. B: Follow-up CT at nine months showing worsening anterior chronic anterior superior endplate compression without the progression of kyphotic angulation at L3. There now is a greater than 50% loss of height of anterior L3 (dashed black arrow). There is a 2 mm posterior displacement of the superior endplate L3 (solid black arrow) and compressed bone surrounding the endplate (dashed black arrow). C: Initial CT scan showing mild-lateral listhesis and 3.9^0 ^of angulation at L4-L5. There are two lateral superior endplate fractures at L3 (dashed white arrow). D: Follow-up CT nine months after onset now showing further collapse but worse laterally toward one side (dashed black arrows). There is the development of vacuum intra-discal changes at L4-L5 (solid black arrow) in the area of lateral listhesis.

Displacement of the posterior part of the fractured endplate into the ventral spinal canal and the recognition of a fracture with a worsening collapse or extending into the posterior wall have been found to be factors that may predict the risk of intra-discal and epidural leakage of cement during VA and KP [20]. CT scans with three plane reconstruction is especially helpful in evaluating the location of a fracture within the vertebra, the displacement of the endplate, and the degree of kyphosis or associated degenerative scoliosis (Figure 2).

**Figure 3:**
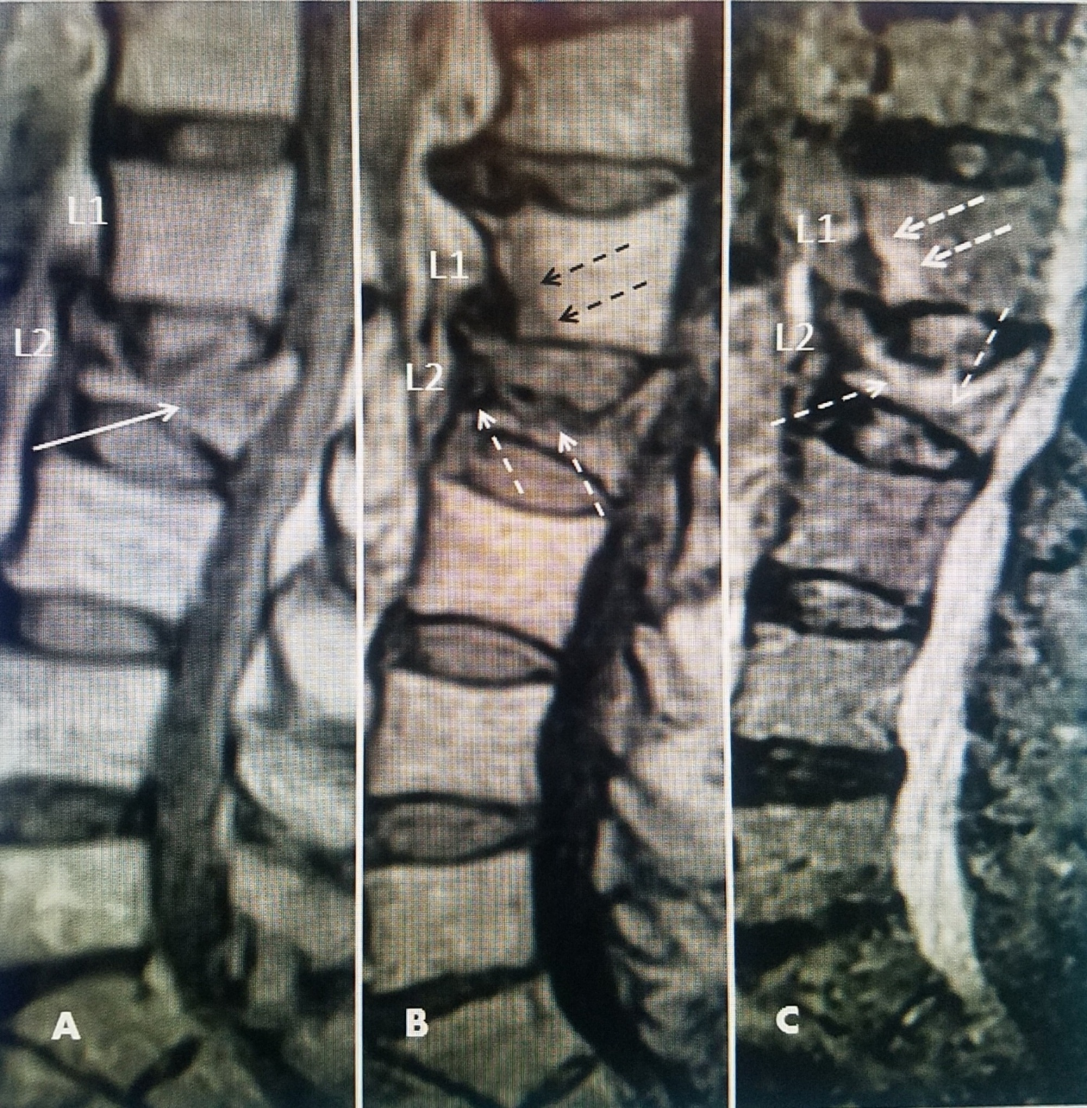
Magnetic resonance imaging (MRI) showing the development of adjacent fractures over seven months A 73-year-old female with a history of multiple falls. She complained of chronic lumbar pain and the original MRI showed an L2 fracture but then developed an L1 adjacent-level fracture over six months despite the use of a brace and anti-inflammatory medication. A: Six weeks after the latest fall, sagittal T1 MRI showing a non-edematous superior endplate fracture at L2 (solid white arrow) with the posterior displacement of the superior endplate into the spinal canal. B; Sagittal T1 MRI taken five months later shows progressive collapse at L2 with slight edema under the superior endplate (dashed white arrows) and new edema in the anterior inferior edge of L1 (dashed black arrows). C: MRI with short TI inversion recovery (STIR) images five months after initial MRI now showing extensive edema involving almost the entire body of L2 (thin dashed white arrow) and a clear image of new edema in the anterior inferior part of L1 (thicker dashed white arrows).

With the persistence of the unhealed fracture, serial MRI scans show vertebral body edema on both T1 and, particularly, T2 sequences and short TI inversion recovery (STIR) images. Vertebral body edema is an especially sensitive sign of an active fracture [21]. Vacuum changes in both the intervertebral disc and endplate on CT and fluid seen in the same region on MRI are closely correlated [19-20]. The development of early signs of edema on MRI, especially at an adjacent vertebra, is indicative of progression and instability with a shifting of the weight load and stress on the next vertebral level. This is a frequent occurrence with chronic fractures (Figure 3).

**Figure 4:**
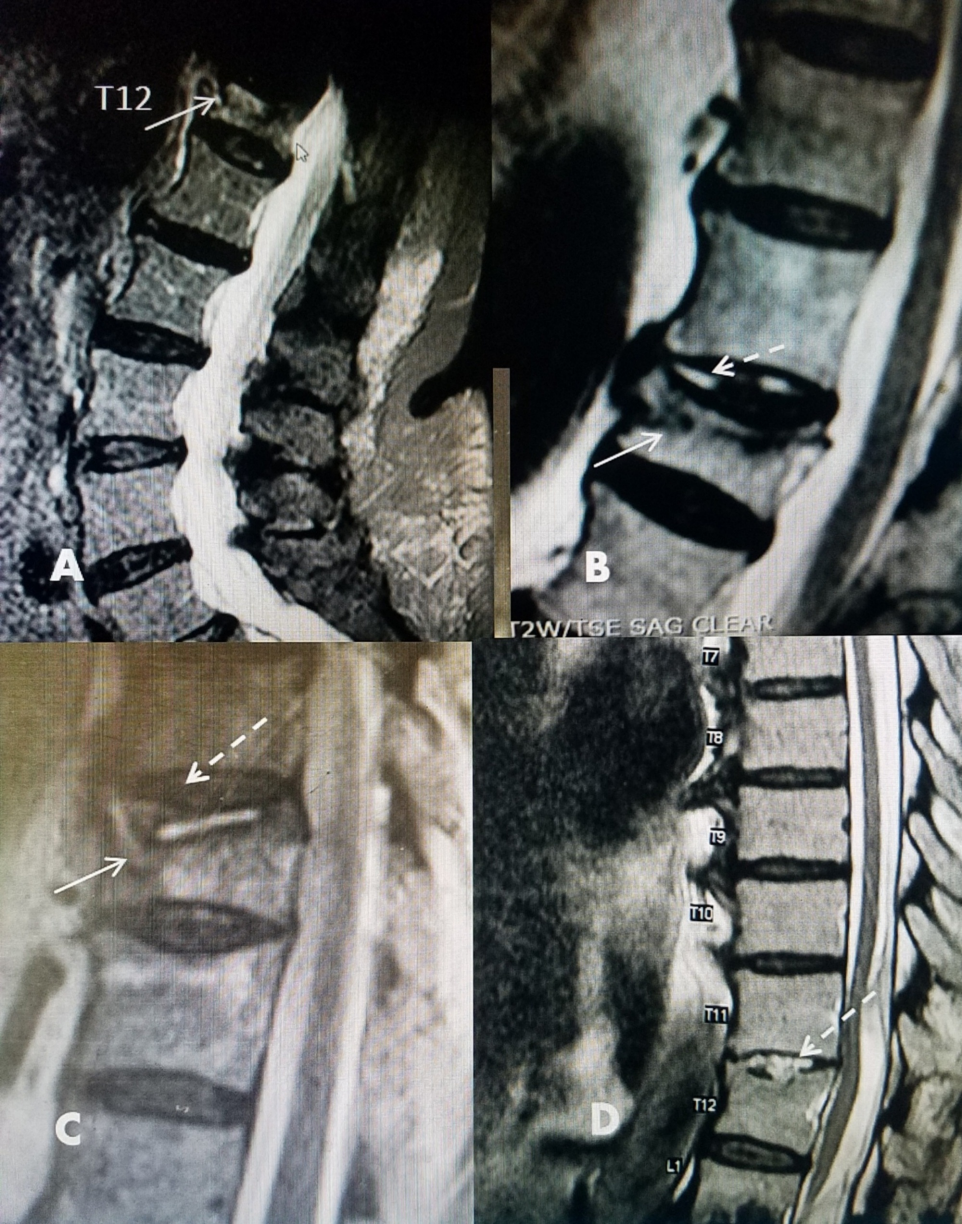
Examples of vertebral clefts with chronic vertebral compression fractures A: Small high-intensity signal cleft (solid white arrow) within the superior collapsed body of T12. B: T2 magnetic resonance imaging (MRI) showing vertebral collapse, edema (solid white arrow), and vertebral cleft in the superior endplate as well as the disc space (dashed white arrow) at L2. There are a broad-based collapse and mild kyphotic angulation. C: Sagittal T2 MRI in a patient with a T11 chronic fracture with an anterior partial vertebral collapse within the body without angulation (solid white arrow). There is a long high-intensity fluid cleft along the middle part of the superior endplate (dashed white arrow). D: T11-T12 high-intensity fluid cleft extending into the disc space just above a small superior endplate fracture in T12 (dashed white arrow). There is slight kyphotic angulation at the T11-T12 level.

Multiple studies have shown a high correlation between the presence of pain and vertebral clefts on MRI, which are thought to be a sign of vertebral instability [21-22]. The finding of a vertebral fluid-filled cleft on the MRI scan, which often correlates with a "vacuum" change on CT scan adjacent to the fractured superior endplate is highly correlated with spinal micromotion and an unhealed fracture [19,22]. Studies show that the filling of the cleft is closely related to both an improvement in pain and the prevention of further progression of the kyphotic deformity. In these cases, the use of KP while filling the cleft may also restore vertebral height and correct angulation [19]. Follow-up radiologic studies that show the development of these vertebral clefts are indicative of non-healing and are dynamic, and the fluid-filled area can change in size, with positions changing from supine to extension. There is some clinical support for this to indicate that the fracture is progressing with further vertebral collapse, deformity, and angulation [21-22] (Figure 4).

Clinical patterns of chronic VCF

Several distinct groups were identified in this study. The largest group of 21 patients had been diagnosed with an acute VCF that was initially treated conservatively, but the patients continued with persistent or worsening pain with or without further vertebral collapse or deformity but did not undergo VA until at least four months after the initial injury and onset of spinal pain. This would be seen more as a failure of conservative treatment [2-3]. Even though VA and KP are usually performed under local anesthesia, it was noted that many were elderly with medical co-morbidities, such as cardiac or pulmonary insufficiency, using anticoagulants for thrombophlebitis, or atrial fibrillation, which led to a delay in medical clearance for the surgery. This occurred in seven of the 21 patients and all had multiple fractures that would require procedures at several levels.

**Figure 5:**
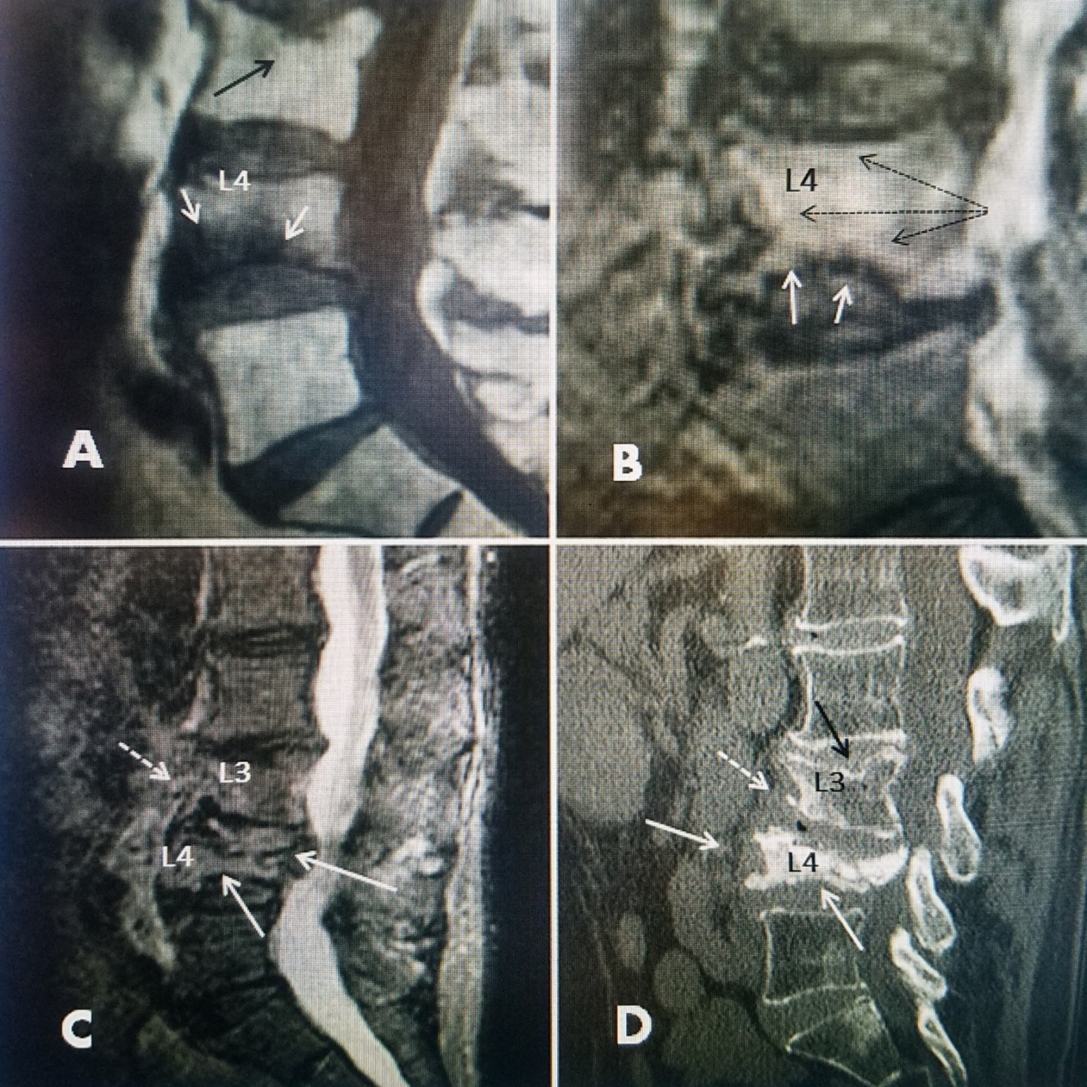
Progressive collapse of an inferior L4 endplate fracture over nine months A: Initial T1 magnetic resonance imaging (MRI) three weeks after a fall, showing a small, inferior endplate fracture at L4 with 50% vertebral body edema (solid white arrows). There is an old, small superior endplate fracture at L3 (solid black arrow). B: The patient used a lumbar support for three months but because of persistent pain, a repeat MRI scan was performed four months after injury and a short TI inversion recovery (STIR) MRI showing almost 100% edema of the L4 vertebral body indicated by a high-intensity uptake extending throughout L4 (dotted black arrows). The main area of the fracture is confined to the inferior endplate (solid white arrows) although the edema is spread in the entire L4 vertebra. Patient refused vertebral augmentation at that time. C: MRI scan seven months after injury now showing the progressive collapse of L4 with the posterior protrusion of the vertebra into the lumbar canal. There is also a "mottled" marrow single in L3 (dashed white arrow), suggesting a new adjacent-level fracture. D: Patient progressed to a "pancake" like collapse. Bilateral vertebral augmentation (VA) was performed to stabilize the fracture and the patient had an 80% reduction in pain after two weeks. However, the patient is developing a new endplate fracture inferiorly at L3 (dashed white arrow). The old superior endplate fracture initially seen when first diagnosed is still apparent (solid black arrow). Biopsy performed at the time of vertebral augmentation (VA) was negative for cancer and only showed severe osteoporotic bone.

In a second group, comprising seven patients, the diagnosis of VCF was delayed because initial attention was focused on another area of the spine or the fracture area was not visualized: two of the four were at the thoracic-lumbar junction and in another two, the original plain X-rays were poor-quality films of obese patients, which did not visualize the fracture The third group consisted of three patients where the initial attention was the lumbar spine and the thoracic-lumbar and thoracic spine films were not obtained until the patient complained of persistent pain in these areas after undergoing therapy for the lumbar spine with a diagnosis of degenerative discs and spondylosis being made after falls or auto accidents. In these cases, the degenerative pathology was initially treated and the VCF was not diagnosed immediately until the persistence of pain or recognition that the pain was in another spinal area, like the lower thoracic spine compared to the lumbar spine, led to the discovery of the fracture on new imaging studies [11-12] (Figure 5).

Elderly patients with osteopenia or osteoporosis can develop spinal pain, usually after a fall or accident, which characteristically is often a minor or low-impact event but can still lead to a VCF [1,3]. The majority of single-level osteoporotic fractures respond to conservative treatment, including a combination of bracing, mild anti-inflammatory, and pain medication, followed by physical therapy and especially back extension exercises. There is a controversy about both the need and timing of performing vertebral augmentation procedures immediately versus delayed for several weeks to give conservative treatments time to be effective [2]. Even with conservative treatment, some patients persist with pain or cannot tolerate bracing and, after an initial two to six weeks, may need to consider interventional treatment, such as vertebral augmentation (VA) or kyphoplasty (KP). Large meta-analysis studies of conservative versus interventional treatment do not show statistically significant differences although elderly patients can be mobilized faster after KP or VA [1]. Follow-up data shows a better restoration of vertebral height and a reduction of the angle of mild kyphotic deformity if these procedures are performed within the initial two to six weeks compared to when it is performed 16 or more weeks after the onset [23-25]. However, especially after balloon kyphoplasty, longer-term follow-up studies have shown loss of the initial height reduction in up to 25%-30% of cases although this is noted on post-procedure radiologic studies, often without any recurrence of clinical pain [26-28]. Patients that had a previous KP were found to have an increased risk of developing adjacent level fractures. This may lead to more chronic spinal pain that is initially attributed to the first treated fracture. Not recognizing the possibility of the development of adjacent level fractures may lead to missed or delayed fracture diagnosis and treatment. This can occur If the patient is not properly re-evaluated with CT or MRI scans prior to the VA procedure [19,27-29].

The use of vertebral augmentation in the treatment of painful chronic fractures

The use of either VA or KP in the treatment of chronic fractures, defined as fractures at least 16 weeks or greater after onset, was initially reported anecdotally soon after the adoption of the procedure in the 1990s although the indication and timing of the procedure were still not clear [1-2,30]. Several early studies reported one to three cases within larger series that treated chronic fractures 12 months but even as late as 36 months after onset, showing that the best results based on pain reduction occurred within six to 24 months from injury [4-6]. In 2001, Kaufmann presented 75 patients with 122 treated fractures with a mean time to treatment of 19 weeks. There were only 10 patients between 12 and 24 months but with good results and VAS scores dropped from 9.4 before to 1.9 after late KP [4]. This study included patients treated from 1995 to 2001 and only 34% of the patients had MRI scans and 50% bone scans for diagnosis so the findings with recent radiology studies demonstrating the importance of both the degree of vertebral edema, endplate collapse, and especially the development of vertebral clefts as related to chronic pain would not have been apparent. Then in 2004, Brown reported 41 patients with 78 treated chronic fractures, with 16 patients treated between 12 and 24 months and 25 patients treated greater than 24 months from injury [5]. There was an 80% improvement with 17% obtaining complete relief from pain. This compared to a control group with more acute and sub-acute fractures that had 92% relief but the difference was not statistically significant. Also in 2004, Crandall reported a study of 47 patients with 55 fractures and compared the results of KP in acute verse chronic fractures [6]. Although the overall improvement in pain measured by VAS was statistically the same at 85%-90%, acute fractures had a better restoration of height and a reduction of kyphosis than chronic fractures. In both studies, when examining chronic fractures, there were better overall results in the 12- to 24-month time frame similar to Kaufmann's earlier work [5-6]. Interestingly, both studies were reported in the American Journal of Neuroradiology but basically focused on reporting the location of the fracture and the effect of the procedure on vertebral height but there was no review of radiologic findings with chronic VCF [4-6]. One of the most important findings that has been recognized with the more common use of MRI scans is the identification of a fluid-filled superior endplate cleft with unstable and chronic fractures and understanding the importance of filling the cleft as well as the entire area under the collapsed endplate to obtain effective late pain relief and prevent further collapse with vertebral augmentation [18,22,30]. Another important factor that has been recognized with follow-up MRI scans in patients with chronic fractures is the development of additional fractures at adjacent levels in the months from injury to treatment often indicated by vertebral body edema in the adjacent vertebra on MRI scan even before collapse occurs [19,21].

In this study, it is apparent that when a patient has localized chronic spinal pain concurrent with a variety of radiologic findings on bone scan, CT, and MRI, demonstrating the existence of a vertebral fracture then either KP or VA is a reasonable treatment option with low risk and a significant chance of pain relief. Specific radiologic findings, such as progressive endplate collapse, possibly persistent uptake on bone scan, edema on MRI scan, and especially the development of fluid clefts or vacuum changes on MRI or CT scan, are strong indicators of unhealed fractures. Persistent pain or incomplete pain relief after conservative treatment does not exclude the possible later treatment with vertebral augmentation as long as 24 months after the onset of the fracture.

## Conclusions

Late vertebral augmentation based on radiologic findings of continued fracture instability and non-healing is effective in patients with chronic fractures between four to as long as 36 months after the initial injury. Radiology studies, especially MRI and reconstructed multiplane CT scans as well as bone scans, are more routinely used today than when the initial studies of chronic fractures were made in 2001 and 2004, making it possible to identify specific imaging findings indicative of continued non-healing or instability with chronic VCF. These radiologic changes include persistent bone scan uptake, MRI edema, the presence of fluid-filled vertebral clefts, and CT findings of vacuum changes in the intervertebral disc and vertebral endplate associated with progressive collapse or kyphosis. These radiologic findings combined with localized pain may indicate a need for vertebral augmentation. In this series of patients, the VAS improved by 66% whether the patient had single or multiple fractures treated. An unanticipated finding was a higher percentage of lumbar and sacral fractures than routinely seen in the normal distribution of VCF. The presentation of persistent lumbar pain in elderly osteoporotic patients may be initially attributed to concurrent or pre-existing lumbar degenerative disease while it may be due to an underlying chronic VCF, leading to delays in diagnosis and, ultimately, the treatment of the VCF.
